# The Gene Ail for the Attachment–Invasion Locus Protein of *Yersinia enterocolitica* Biotype 1A Strains Is Located on the Genomes of Novel Prophages

**DOI:** 10.3390/ijms262211166

**Published:** 2025-11-19

**Authors:** Jens Andre Hammerl, Stefan Hertwig

**Affiliations:** Consultant Laboratory for Yersinia, Department Biological Safety, German Federal Institute for Risk Assessment, 10589 Berlin, Germany; jens-andre.hammerl@bfr.bund.de

**Keywords:** *ail*, virulence, *Yersinia enterocolitica*, prophage, horizontal gene transfer

## Abstract

The attachment–invasion locus protein Ail of pathogenic *Yersinia* strains is an important virulence factor, both for invasion of eucaryotic cells and for serum resistance. In other *Yersinia* strains, e.g., those belonging to biotype (BT) 1A of *Yersinia enterocolitica*, *ail* has only occasionally been described. Sequence analysis of 370 BT 1A isolates in our laboratory revealed 41 (11.1%) which were *ail*-positive. Most of these isolates were recovered from minced meat and tonsils of wild boars, and belonged to 17 MLST allele profiles. A closer look at DNA sequences surrounding *ail* disclosed that the gene in most isolates is embedded in DNA regions encoding phage proteins. The genomes of four prophages belonging to four different phylogenetic clusters were determined and analyzed by in silico studies. These have sizes of 34.9 and 50.7 kb, and are closely related to each other but not to known phages. Unlike other regions of the prophages, the integrases and attachment sites of some of them diverge, leading to different integration sites in the isolates. In a fifth cluster, *ail* is relocated at a position on the *Y. enterocolitica* chromosome that is several hundred kilobases apart from those of the other clusters, but surrounded by prophage-related sequences. In addition, highly pathogenic 1B/O:8 strains contain a DNA segment which includes *ail* and is 65 to 94% identical to the prophage sequences determined in this study.

## 1. Introduction

Yersiniosis is an infectious disease of the gastrointestinal tract caused by *Yersinia* (*Y.*) *enterocolitica* and, to a lesser extent, by *Y. pseudotuberculosis* [1,2]. It is the third-most common bacterial enteritis in Europe. Infections are mainly caused by the consumption of raw or undercooked pork [3,4,5,6,7,8,9]. *Y*. *enterocolitica* comprises six biotypes (BT) and up to 70 serotypes. While the BTs 1B, 2, 3, 4 and 5 possess the 70 kb virulence plasmid pYV encoding a type III secretion system and effector proteins (*Yersinia* outer proteins = YOPs) with partly toxic activity for eucaryotic cells, BT 1A strains are generally devoid of pYV [10]. Moreover, some important chromosomally encoded virulence factors of pathogenic biotypes exist only rarely or not at all in BT 1A [11,12]. Two of those are the enterotoxin YstA and the attachment–invasion locus protein (Ail), the latter being involved in both invasion of eucaryotic cells and serum resistance [13,14,15]. To date, the *ail* gene has only been detected in some BT 1A strains [16,17]. Because of the lack of these virulence factors, BT 1A strains have for a long time been considered to be non-pathogenic. However, in recent years, there have been an increasing number of reports describing BT 1A isolates from clinical cases [18,19,20,21,22,23,24,25,26]. This biotype obviously comprises at least two phylogenetic lineages, each with different virulence factors, some of which are toxins [17,27]. The question arises as to whether horizontal gene transfer may be involved in the heterogeneity of this biotype. In this study, we sequenced 370 BT 1A isolates from different sources. Among these isolates, 41 contained the virulence gene *ail*. A closer analysis of this gene revealed that, unlike in the strictly pathogenic biotypes, *ail* in most BT 1A isolates is located on the genomes of prophages. These prophages were then characterized by in silico analyses.

## 2. Results

### 2.1. Out of 370 BT 1A Genomes, 41 Contain a Prophage-Associated Ail Gene

**Figure 1:**
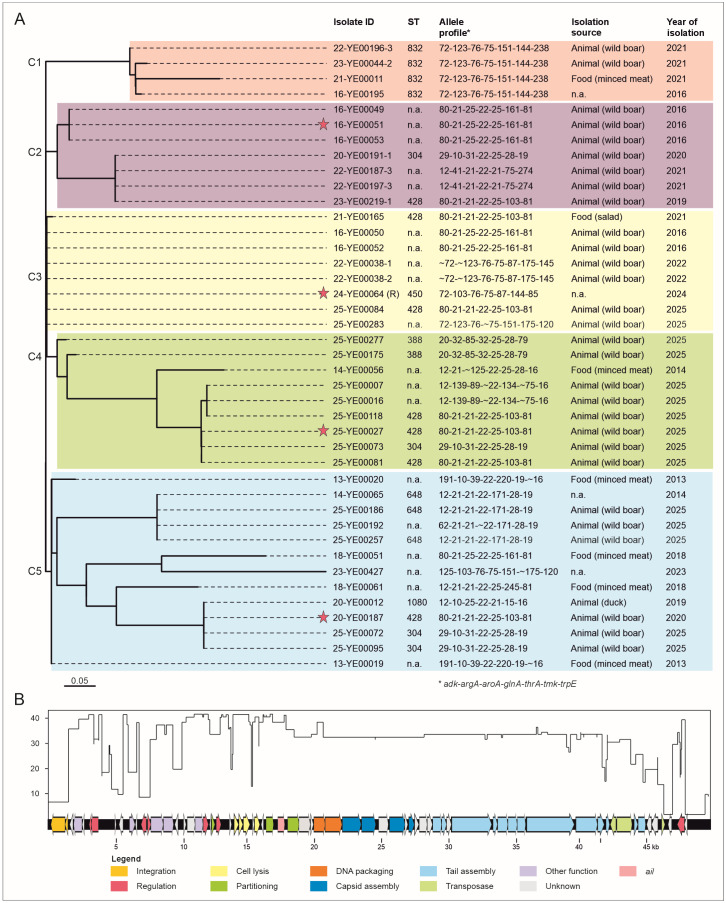
Relationships among prophages containing an *ail* gene. (**A**) Clusters of similar prophage genomes in *Y. enterocolitica* BT 1A isolates, obtained by comparison with the vB_Yen_24-YE00064 prophage (R, reference genome) of cluster 3. For better visualization, the individual clusters are colored. The sequence types (STs) of their hosts, as well as the source and year of isolation, are stated. Red stars indicate isolates in which whole-*ail* prophage sequences were determined. Phylogenetic evaluation was conducted using vB_Yen_24-YE00064 prophage as reference genome for the comparison with WGS sequencing data of *ail*-gene-carrying *Y. enterocolitica* BT 1A isolates (Bioproject no. PRJNA1347376). CSI phylogeny (version 1.4) was used for reference-based SNP-tree generation using default parameter settings. The scale bar represents a reference for the number of nucleotide substitutions per site. (**B**) Conserved DNA regions of the prophage genomes. The plot gives the numbers of related phage sequences identified in the compared WGS dataset of the isolates (y-axis). On the x-axis, the conservation of specific DNA regions of the vB_Yen_24-YE00064 prophage is given. The map shows the genome of the prophage vB_Yen_24-YE00064 used as reference for sequence comparison (Table S2).

Sequence analyses of 370 *Y. enterocolitica* BT 1A isolates revealed that 41 of them harbor the gene *ail*. Isolates were obtained between 2013 and 2025 from different sources (mainly minced meat and wild boars). These isolates belonged to 17 different MLST allele profiles (Figure 1A). All *ail*-positive isolates (n = 41) possessed the enterotoxin gene *ystB*, while thirty, ten and three isolates additionally possessed the virulence-associated genes *hreP*, *myfA* and *tccC*, respectively (Table S1). For 39 isolates, plasmid-borne sequences of approximately 3 to 106 kb were predicted.

A closer look at the *ail* gene in these isolates showed that it is up to 99% identical to the *ail* in pathogenic biotypes, e.g., the bio/serotype 1B/O:8 strain 8081. In addition, the upstream sequence containing the promoter and ribosome-binding sites of *ail* is similarly related to its counterpart in other biotypes, suggesting that *ail* may be active in BT 1A.

However, the regions encompassing *ail* diverge significantly in the various biotypes. In most BT 1A isolates, such as the 24-YE00064 studied here, the gene is surrounded by partitioning genes and genes for cell lysis (lysin and holin), as well as DNA packaging (small and large terminase) typically associated with phage (Table S2). Moreover, genes for phage assembly (capsid and tail) and for genetic switch, as well as integrase and excisionase, were also identified in 37 chromosomes, suggesting that *ail* is part of a prophage (Table S2).

### 2.2. Comparison of the Prophages Indicates Relationships Between Them

**Figure 2:**
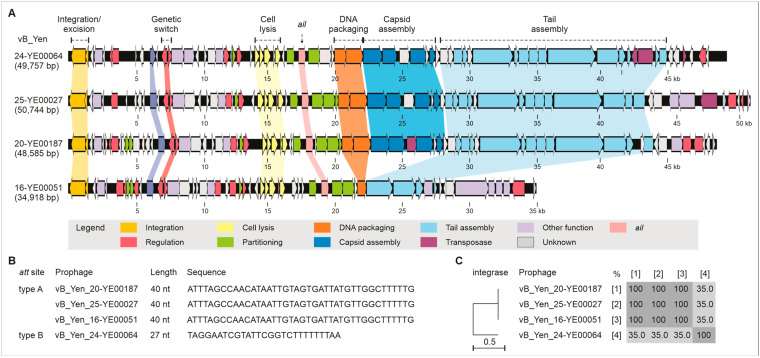
Genome organization and similarities of the integrases and attachment sites of the four analyzed prophages. (**A**) Genome maps of the prophages. The genes are colored according to their functional prediction as specified in the figure legend. The relationship between the main functional gene clusters of the prophages is indicated. Sizes of genomes and predicted functions of assigned ORFs are given. (**B**) Sequences of the attachment sites of the prophages. (**C**) Similarities of their integrases.

The 41 prophage sequences identified in the investigated isolates by comparison with the vB_Yen-24-YE00064 prophage form five major clusters (Figure 1A). Among these, the prophages in the clusters C2 to C5 harbor the *ail* gene, whereas prophages in cluster C1 are devoid of *ail* because here the gene is located at a different position on the chromosome (see below). An alignment disclosed that most regions of the prophages, particularly those encoding structural proteins, are closely related (up to 85%), whereas the integrase genes show major differences (Figure 1B). Short-read sequencing allowed the prediction of four whole-prophage genomes (vB_Yen_16-YE00051, vB_Yen_20-YE00187, vB_Yen_24-YE00064 and vB_Yen_25-YE00027) belonging to four clusters (Figure 1A). These have genome sizes between 34,918 and 50,744 bp, and are composed of between 55 and 78 Open Reading Frames (ORFs, Table S2). The overall genome organization of the prophages is similar (Figure 2A). As with other temperate phages, ORFs for repressor proteins, cell lysis, DNA packaging, and capsid and tail assembly are clustered, even though some ORFs, particularly those encoding structural proteins, are missing in vB_Yen_16-YE00051. The prophages showed no identities to other phages, and only some relatedness to two *Y. enterocolitica* BT 1A chromosomes (Y201, CP124238.1; and Y115, CP124259.1).

Three prophages (vB_Yen_16-YE00051, vB_Yen_20-YE00187 and vB_Yen_25-YE00027) have an identical attachment site, *att*, of 40 bp (Figure 2B). The integrases of these prophages are 100% identical (Figure 2C). By contrast, the prophage vB_Yen_24-YE00064 has an *att* site of only 27 bp (Figure 2B). The integrase of this prophage is only approximately 35% identical to those of the other ones (Figure 2C). Thus, it is not surprising that the two groups have different integration sites on the bacterial chromosome. While the prophages vB_Yen_16-YE00051, vB_Yen_20-YE00187 and vB_Yen_25-YE00027 are integrated between two genes for hypothetical proteins, vB_Yen_24-YE00064 is integrated between a gene for an integrase and a YebY family protein.

### 2.3. Relocation of Ail in Cluster C1 and Analysis of 1B/O:8 Strains

**Figure 3:**
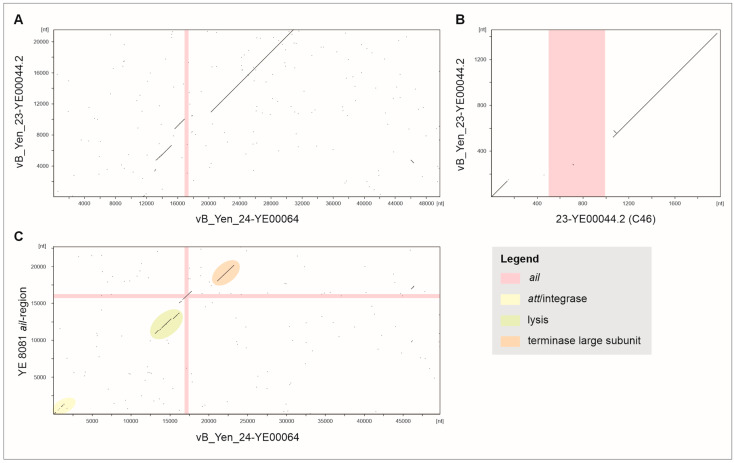
Dot plots of the prophages vB_Yen_24-YE00064, vB_Yen_23-YE0044.2 and the ØYE200 region of strain 8081. DNA sequence comparison was conducted using DS Gene (version 2.5, Accelrys Inc., San Diego, CA, USA) by using NA identity matrix for scoring with the following parameter values: window size = 30; hash value = 6; minimal score = 65%. DNA sequences used for nucleotide comparison are given as labels for x- and y-axes. Black lines indicate DNA regions with similarity values of >65% between the sequences. (**A**) The vB_Yen_24-YE00064 *ail* prophage is similar to the cluster C1 prophage vB_Yen_23-YE0044.2 lacking *ail*. (**B**) In isolate 23-YE00044.2, *ail* is located on a different contig (C46) to the prophage but is surrounded by prophage-related sequences, as indicated in red. The dot plot shows only a small part of the conserved part of vB_Yen_23-YE0044.2 which is similar to the *ail* region. (**C**) vB_Yen_24-YE00064 is related to the ØYE200 prophage region of strain 8081, which also includes *ail* and an abridged terminase large subunit gene. Colors indicate functional assignments of the related sequences as specified in the figure legend.

Sequence analysis of cluster C1 revealed similar prophage sequences as in isolate 24-YE00064. However, at the position of *ail* in vB_Yen_24-YE00064, there is a gap in the C1 prophage genome (Figure 3A). In this cluster, *ail* is located approximately 600 kb apart from the vB_Yen_24-YE00064-related prophage sequences. Interestingly enough, the gene and its adjacent sequences are surrounded by DNA segments which are similarly present in the corresponding prophages, as shown for the prophage vB_Yen_23-YE00044.2 (Figure 3B). The homologous upstream and downstream sequences of *ail* have lengths of 144 bp and 925 bp, respectively, but are not related to each other. Therefore, it remains open how *ail* was relocated in isolates belonging to cluster C1. Nevertheless, it is noteworthy that even in the highly pathogenic *Y. enterocolitica* 1B/O:8 strains 8081 (AM286415.1), WA (CP009367.1) and Billups-1803-68 (CP173224.1), *ail* is associated with phage genes. Indeed, a stretch of approximately 20 kb of strain 8081 containing *ail* is similar to vB_Yen_24-YE00064 (Figure 3C). This stretch essentially corresponds to the ØYE200 region identified in 8081 [28]; this, however, has been determined to be a smaller prophage (15.5 kb) without *ail*. Besides *ail*, the 20 kb prophage of strain 8081 comprises genes for an integrase, lysis proteins (holin and lysin) and the terminase large subunit. Moreover, the fact that this DNA segment also contains the 27 bp attachment site of vB_Yen_24-YE00064 upstream of the integrase gene, and that this site is linked to tRNA genes, suggests that *ail* was once associated with a similar prophage. It is conspicuous that the DNA segment in strain 8081 harbors several transposase genes which are lacking in vB_Yen_24-YE00064 and which might have been involved in genetic reassortments.

## 3. Discussion

The attachment–invasion locus protein Ail of *Y. enterocolitica* is an important virulence factor which is produced by all pathogenic biotypes of this species, as well as by *Y. pseudotuberculosis* and *Y. pestis* [29]. To date, it has only rarely been described in BT 1A strains of *Y. enterocolitica* and in *Y. enterocolitica*-like species, e.g., *Y. kristensenii,* some of which have been reported to contain an additional *ail*-related gene which may be associated with plasmids or phages [11,30,31]. For this reason, *ail* is routinely used as a target in detection of pathogenic *Y. enterocolitica* and *Y. pseudotuberculosis* by RT-PCR (ISO TS 18867:2015). However, sequencing of 370 BT 1A isolates showed that *ail* is present more commonly than expected in this biotype. We identified the gene in 41 (11.1%) out of 370 isolates recovered from food and wild boars in the last 12 years. The *ail*-positive isolates represent a broad range of MLST allele profiles, even though some types (ST304, ST428, ST832) were prevalent. The question arises as to how BT 1A strains may acquire *ail*. This study suggests that it may occur by lysogenic conversion via temperate phages. Indeed, the analysis of *ail*-positive BT 1A isolates showed that in most of them the gene is located on a prophage. The prophages are related to each other, but form five different clusters. Up to now, four prophage genomes containing *ail* could be analyzed in detail. The analysis suggests that three of them (vB_Yen_20-YE00187, vB_Yen_24-YE00064 and vB_Yen_25-YE00027) may be active, because all elements required for the formation of a phage particle are obviously present, whereas the prophage vB_Yen_16-YE00051 is presumably defective because some essential ORFs for capsid and tail proteins are lacking. Indeed, initial induction experiments with an isolate (18-YE00061) belonging to cluster C5 demonstrated that, like other prophages of this isolate, the *ail* prophage is inducible and forms plaques on some *ail*-negative BT 1A strains, though only at 37 °C (to be published elsewhere). The fact that genes for structural proteins of the complete prophages are very similar indicates that the corresponding phage particles may have the same morphology. A striking difference, however, pertains to their integration site on the *Y. enterocolitica* chromosome. Unlike some other parts of their genomes, genes for the integrase and attachment sites are in part highly diverse. As a consequence, the prophages are not integrated at the same position in BT 1A strains. Whether these sites also exist in other *Y. enterocolitica* biotypes or even other *Yersinia* species, and whether those strains may also acquire *ail* by phage-mediated transfer, has still to be studied. It has to be taken into account that to achieve lysogenic conversion the host range of a temperate phage is of major importance. *Yersinia enterocolitica* BT 1A strains belong to various serotypes, which may determine the host specificity of a phage. It appears, however, that *ail* prophages are subjected to genetic recombination. This can be clearly seen in cluster C1, comprising related prophages, in which *ail* including adjacent sequences has been relocated. Similarly, highly pathogenic 1B strains like 8081 contain remnants of *ail* prophages, suggesting recombination events or even a horizontal gene transfer in the past. Thus, it is conceivable that a related temperate phage of *Y. enterocolitica* 1B/O:8, which has already been isolated from wild boars in Europe [32], once infected, say, a BT 1A/O:8 strain, followed by recombination with an indigenous prophage. Whether such an event has a selective advantage for BT 1A is not clear. However, it is noteworthy that most isolates of this study were recovered from tonsils of wild boars, where the presence of an *ail* gene might be beneficial for survival. Because wild boars may come into contact with other animals (e.g., pigs) and can contaminate vegetables and salad plants growing in fields, it is possible that *ail*-positive *Y. enterocolitica* isolates obtain access to the food chain. Indeed, the fact that some of the *ail*-prophage-containing isolates were recovered from minced meat indicates that they were lysogenized with such a phage. This means that the spread of *ail* in pig farms cannot be ruled out. However, it has still to be demonstrated that Ail is really produced by BT 1A, even though this does seem likely due to the similarity of its *ail* and upstream sequences to those of pathogenic biotypes. Regrettably, information on the prevalence of temperate *Y. enterocolitica* phages and their potential to exchange genes is still scarce [33,34]. Popp et al. (2000) isolated eight temperate phages from 170 *Yersinia* strains by induction with mitomycin C [34]. Seven phages were isolated from *Y. enterocolitica*, most of which belonged to BT 1A. Some phages were able to infect pathogenic and non-pathogenic *Y. enterocolitica* strains. It was shown that one of those phages (PY20) isolated from a B4/O:3 strain was able to transduce small *Yersinia* plasmids, but not the 70 kb virulence plasmid pYV [35]. In another study, ten temperate phages were isolated from 102 pathogenic bio/serotypes of *Y. enterocolitica,* some of which additionally lysed BT 1A [36]. They formed plaques exclusively at room temperature. However, data on the potential of these phages to transduce DNA have not been published. We will therefore extend our study on temperate phages to investigate whether lysogenic conversion is involved in the spread of the *ail* gene.

## 4. Materials and Methods

### 4.1. Source and Typing of Y. enterocolitica Strains

The 370 isolates analyzed in this study are part of our *Yersinia* strain collection. This collection comprises both isolates from food sent by local laboratories to the BfR (German Federal Institute for Risk Assessment) for typing and isolates from prevalence studies on *Yersinia* in wild boars carried out between 2019 and 2025. Here, tonsils of wild boars, shot in the north-east of Germany, were examined for the presence of *Yersinia* using the EN ISO method 10273:2017 or an improved version of it [37]. The isolates were cultivated on Columbia agar supplemented with 5% sheep blood (bioMérieux Deutschland GmbH, Nürtingen, Germany) at 28 °C for 16–20 h for whole-cell matrix-assisted laser-desorption/ionization time-of-flight mass spectrometric identification (MALDI-TOF MS) using the direct transfer method with HCCA matrix on a Biotyper (Bruker Daltonics GmbH and Co. KG, Bremen, Germany). In addition, physiological and biochemical tests using classical tube and plate procedures for species confirmation and biochemical differentiation were conducted as previously described [38]. Unless otherwise indicated, YP cultivation was conducted at aerobic conditions at 28 °C for 18–24 h using lysogeny broth (LB)-based media. For solid media preparation, LB medium was supplemented with 1.8% Bacto Agar No. 1 (Oxoid Deutschland GmbH, Wesel, Germany) [39,40].

### 4.2. Genome Sequencing and Bioinformatics Analysis

Whole-genome sequencing (WGS) of *Y. enterocolitica* BT 1A isolates was performed by short-read, paired-end sequencing (2 × 150 cycles) on a NextSeq500 benchtop device (Illumina Inc., San Diego, CA, USA). Bacterial genomic DNA was extracted from liquid cultures grown at 28 °C for 20–24 h using the PureLink Genomic DNA Mini Kit (Invitrogen, Ebersberg, Germany) according to the recommendation of the manufacturers. DNA sequencing libraries were prepared using the Nextera XT DNA Sample Preparation Kit (Illumina Inc.) [33,36,41,42]. Raw sequencing data were subjected to the Aquamis (v1.4.1) pipeline [43] for quality evaluation, demultiplexing and trimming, while general in silico typing tasks were conducted using BakCharak (https://gitlab.com/bfr_bioinformatics/bakcharak; accessed on 15 July 2025). The BakCharak pipeline (v3.1.6) includes tools for prediction of MLST sequences (pubMLST) and for detection of antimicrobial resistances (AMRfinder [v3.12.8], NCBI resistance gene database [v2024-01-31.1]), plasmids (Abricate [v1.0.1], plasmidfinder [v2.0.1] database [v2022-07-13]), virulence factors (Abricate [v1.0.1], VFDB database [v2024-06-25]), and close reference genomes ((mash [v2.3]), NCBI refseq, plasmid reference finder (mash [v2.3]), NCBI plasmid database [v2.14.1]). In addition, BakCharak was used for plasmid comparison (Blastn [v2.14.1], NCBI plasmid database (accessed on 3 September 2025)) and fastANI species prediction [v1.33] (NCBI refseq database [v2021-11-19]) [43]. Prophage detection was conducted using the Phastest tool (v3.0) for initial screening, while manual curation using Accelrys DS Gene (v2.5; Accelrys Inc., San Diego, CA, USA) was performed to determine the complete prophage sequence from attachment sites of bacteria (*attB*) and prophages (*attP*). Genome annotation of the prophage genomes was conducted using the Bacterial and Viral Bioinformatics Resource Center (BV-BRC, v3.54.6). Initial functional prediction of open reading frames (ORFs) was manually curated according to predicted functions of closely related protein sequences derived from blastp searches at NCBI (National Center for Biotechnology Information) [33,36,41,42]. Phylogenetic relationship of vB_Yen_24-YE00064 (reference) to *Y. enterocolitica* genome datasets encoding Ail were conducted using CSI phylogeny (v1.4; default settings; https://cge.food.dtu.dk/services/CSIPhylogeny/; accessed on 20 September 2025). The software identifies, filter, and validate SNPs from the de novo assembled contigs of *Y. enterocolitica* BV 1A genomes to construct a phylogenetic tree based on concatenated SNP profiles using default settings [44]. Unless stated otherwise, all software tools are used with default settings. Figures were generated using CorelDraw (v13).

### 4.3. Genome Accession Numbers

Deposition of prophage genomes at NCBI Genbank was conducted using BankIt for the prophages vB_Yen_24-YE00064 (PV779719), vB_Yen_16-YE00051 (PX531504), vB_Yen_20-YE00187 (PX109664) and vB_Yen_25-YE00027 (PX109665). The genomes of all *Y. enterocolitica* BV 1A mentioned in Figure 1A and Table S1 are accessible under Bioproject PRJNA1347376.

## Data Availability

The original data presented in the study are openly available in Genbank under accession numbers PV779719 (vB_Yen_24-YE00064), PX531504 (vB_Yen_16-YE00051), PX109664 (vB_Yen_20-YE00187) and PX109665 (vB_Yen_25-YE00027). Genome assemblies of ail-gene-carrying *Y. enterocolitica* BV 1A genomes are accessible under Bioproject PRJNA1347376.

## Supplementary Materials

The following supporting information can be downloaded at: https://www.mdpi.com/article/10.3390/ijms262211166/s1, Table S1: In silico typing features of *Y. enterocolitica* genomes analyzed in this study; Table S2: Annotation of the prophage genomes vB_Yen_24-YE00064, vB_Yen_25-YE00027, vB_Yen_20-YE00187 and vB_Yen_16-YE00051.
